# Times to key events in Zika virus infection and implications for blood donation: a systematic review

**DOI:** 10.2471/BLT.16.174540

**Published:** 2016-11-01

**Authors:** Justin Lessler, Cassandra T Ott, Andrea C Carcelen, Jacob M Konikoff, Joe Williamson, Qifang Bi, Lauren M Kucirka, Derek AT Cummings, Nicholas G Reich, Lelia H Chaisson

**Affiliations:** a Johns Hopkins Bloomberg School of Public Health, 615 North Wolfe Street, Baltimore, Maryland, MD 21205, United States of America (USA).; b Johns Hopkins University School of Medicine, Baltimore, USA.; c University of Florida, Gainesville, USA.; d School of Public Health and Health Sciences, University of Massachusetts Amherst, Amherst, USA.

## Abstract

**Objective:**

To estimate the timing of key events in the natural history of Zika virus infection.

**Methods:**

In February 2016, we searched PubMed, Scopus and the Web of Science for publications containing the term *Zika*. By pooling data, we estimated the incubation period, the time to seroconversion and the duration of viral shedding. We estimated the risk of Zika virus contaminated blood donations.

**Findings:**

We identified 20 articles on 25 patients with Zika virus infection. The median incubation period for the infection was estimated to be 5.9 days (95% credible interval, CrI: 4.4–7.6), with 95% of people who developed symptoms doing so within 11.2 days (95% CrI: 7.6–18.0) after infection. On average, seroconversion occurred 9.1 days (95% CrI: 7.0–11.6) after infection. The virus was detectable in blood for 9.9 days (95% CrI: 6.9–21.4) on average. Without screening, the estimated risk that a blood donation would come from an infected individual increased by approximately 1 in 10 000 for every 1 per 100 000 person–days increase in the incidence of Zika virus infection. Symptom-based screening may reduce this rate by 7% (relative risk, RR: 0.93; 95% CrI: 0.89–0.99) and antibody screening, by 29% (RR: 0.71; 95% CrI: 0.28–0.88).

**Conclusion:**

Neither symptom- nor antibody-based screening for Zika virus infection substantially reduced the risk that blood donations would be contaminated by the virus. Polymerase chain reaction testing should be considered for identifying blood safe for use in pregnant women in high-incidence areas.

## Résumé

**Objectif:**

Estimer le chronométrage des événements clés de l'histoire naturelle de l'infection à virus Zika.

**Méthodes:**

En février 2016, nous avons recherché dans PubMed, Scopus et Web of Science des publications contenant le terme *Zika*. À l'aide des données rassemblées, nous avons estimé la période d'incubation, le laps de temps avant la séroconversion et la durée de l'excrétion virale. Nous avons estimé le risque que les dons de sang soient contaminés par le virus Zika.

**Résultats:**

Nous avons sélectionné 20 articles sur 25 patients atteints d'une infection à virus Zika. La période d'incubation moyenne pour l'infection était estimée à 5,9 jours (intervalle de crédibilité, IC, à 95%: 4,4–7,6), 95% des individus ayant développé des symptômes 11,2 jours (IC à 95%: 7,6–18,0) après l'infection. En moyenne, la séroconversion a eu lieu 9,1 jours (IC à 95%: 7,0–11,6) après l'infection. Le virus était détectable dans le sang pendant 9,9 jours (IC à 95%: 6,9–21,4) en moyenne. Nous avons estimé qu’en l'absence de dépistage, le risque qu'un don de sang provienne d'un individu infecté augmentait d'environ 1 pour 10 000 pour chaque augmentation de 1 pour 100 000 personnes–jours dans l'incidence de l'infection à virus Zika. Le dépistage fondé sur les symptômes peut réduire ce taux de 7% (risque relatif, RR: 0,93; IC à 95%: 0,89–0,99) et le dépistage des anticorps, de 29% (RR: 0,71; IC à 95%: 0,28–0,88).

**Conclusion:**

Dans le cas de l'infection à virus Zika, ni le dépistage fondé sur les symptômes, ni le dépistage des anticorps ne permet de réduire considérablement le risque que les dons de sang soient contaminés par le virus. Un test basé sur l'amplification en chaîne par polymérase doit être considéré pour identifier le sang pouvant être utilisé sans danger pour les femmes enceintes dans les zones à forte incidence.

## Resumen

**Objetivo:**

Estimar el cronograma de los eventos clave en la historia natural de la infección por el virus de Zika.

**Métodos:**

En febrero de 2016, se realizaron búsquedas en PubMed, Socups y la Web of Science en busca de publicaciones que incluyeran el término *Zika*. Agrupando datos, se estimó el periodo de incubación, el momento de la seroconversión y la duración de la excreción del virus. Se estimó el riesgo de donaciones de sangre contaminadas con el virus de Zika.

**Resultados:**

Se identificaron 20 artículos sobre 25 pacientes infectados por el virus de Zika. El periodo medio de incubación de la infección se estimó a 5,9 días (intervalo creíble, ICr, del 95%: 4,4–7,6), y el 95% de las personas que desarrollaron síntomas lo hicieron 11,2 días (ICr del 95%: 7,6–18,0) tras la infección. De media, la seroconversión se produjo 9,1 días (ICr del 95%: 7,0-11,6) tras la infección. El virus se podía detectar en la sangre durante 9,9 días (ICr del 95%: 6,9–21,4) como promedio. Sin exámenes, el riesgo previsto de que una donación de sangre proviniera de un individuo infectado aumentó a, aproximadamente, 1 de cada 10 000 por cada incremento de 1 de cada 100 000 personas por día en la incidencia de la infección por el virus de Zika. Las pruebas basadas en síntomas podrían reducir esta tasa en un 7% (riesgo relativo, RR: 0,93; ICr del 95%: 0,89–0,99) y las pruebas de anticuerpos en un 29% (RR: 0,71; ICr del 95%: 0,28–0,88).

**Conclusión:**

Ni las pruebas basadas en síntomas ni las de anticuerpos en busca de una infección por el virus de Zika redujeron considerablemente el riesgo de que las donaciones de sangre estuviesen contaminadas por el virus. Debería realizarse la prueba de reacción en cadena de la polimerasa para identificar la seguridad sanguínea para su uso en mujeres embarazadas en zonas de altos niveles de incidencia.

## ملخص

**الغرض:**

تقدير أوقات وقوع الأحداث الرئيسية في التاريخ الطبيعي لعدوى فيروس زيكا.

**الطريقة:**

قمنا في شهر شباط/فبراير من عام 2016 بالبحث في قواعد البيانات PubMed، و Scopus، و Web of Science للعثور على مطبوعات تتضمن المصطلح " *زيكا *". لقد قمنا من خلال تجميع البيانات بتقدير فترة الحضانة، والوقت المستغرق للتحويل المصلي، ومدة إفراز الفيروس. كما قمنا بتقدير المخاطر الناشئة عن التبرعات بالدم الملوّث بفيروس زيكا.

**النتائج:**

قمنا بتحديد 20 مقالة حول 25 مريضًا مصابًا بعدوى فيروس زيكا. وتم تقدير فترة حضانة الوسيط الخاصة بالعدوى ببلوغها 5.9 أيام (بنسبة ثقة مقدارها ‏95%: 4.4–7.6)، مع وجود 95% من الأشخاص الذين ظهرت عليهم هذه الأعراض في غضون 11.2 يومًا (بنسبة ثقة مقدارها ‏95%: 7.6–18.0) بعد الإصابة بالعدوى. وقد حدث التحويل المصلي في المتوسط بعد مرور 9.1 أيام (بنسبة ثقة مقدارها ‏95%: 7.0-11.6) بعد الإصابة بالعدوى. كان بالإمكان اكتشاف وجود الفيروس بالدم لمدة 9.9 أيام (بنسبة ثقة مقدارها 95‏%: 6.9–21.4) في المتوسط. ومن دون إجراء التحاليل الطبية، ارتفعت نسبة الاختطار المقدّرة للإصابة بالعدوى الناشئة من التبرع بالدم من شخص مصاب بالفيروس بزيادة بلغت 1 في 10000 لكل 1 من بين 100000 شخص–أيام وذلك في معدل وقوع العدوى بفيروس زيكا. قد تؤدي التحاليل القائمة على أعراض الإصابة إلى تقليل هذا المعدل بنسبة ‏7% (الاختطار النسبي، RR: 0.93؛ بنسبة ثقة مقدارها 95‏%: 0.89–0.99) وتحليل الأجسام المضادة بنسبة ‏29% (الاختطار النسبي: 0.71؛ بنسبة ثقة مقدارها ‏95%: 0.28–0.88).

**الاستنتاج:**

لم يقم التحليل القائم على أعراض الإصابة ولا التحليل القائم على الأجسام المضادة لعدوى فيروس زيكا بتقليل نسبة الاختطار من تعرض التبرعات بالدم إلى التلوث بالفيروس بشكلٍ كبير. ويجب مراعاة إجراء اختبار تفاعل البوليميراز المتسلسل لتحديد عينات الدم الآمنة للاستخدام في الحوامل المتواجدات في المناطق التي تعاني من ارتفاع نسب الإصابة بالعدوى.

## 摘要

**目的:**

旨在评估在寨卡病毒感染的自然发展过程中关键事件的时间。

**方法:**

2016 年 2 月，我们在 PubMed、Scopus 及 Web of Science 上检索了包含术语*寨卡*的出版物。 通过整合数据，我们评估了病毒的潜伏期、血清转化时间和病毒传播周期。 我们评估了因献血感染寨卡病毒的风险。

**结果:**

我们确定了 20 篇文章中 25 名感染寨卡病毒的患者。 据估计，出现症状的平均潜伏周期为 5.9 天（95% 置信区间，CrI: 4.4–7.6），感染后，95% 的患者在 11.2 天内 (95% CrI: 7.6-18.0) 出现症状。 平均而言，感染后 9.1 天内 (95% CrI: 7.0–11.6) 发生血清转化。 平均而言，9.9 天后能够在血液中检测到病毒 (95% CrI: 6.9–21.4)。 据估计，因未经筛查受感染个体导致的献血感染病毒的风险为大约每 10 000 天增加 1 例–每 100 000 人中 1 例寨卡病毒感染。 基于症状的筛查能够将此风险降低 7% (相对风险 (RR)：0.93; 95% CrI: 0.89–0.99)  以及抗体筛查降低 29% (RR: 0.71; 95% CrI: 0.28–0.88)。

**结论:**

无论是基于症状的筛查还是基于抗体的筛查，都无法显著降低因献血导致感染此病毒的风险。 在病毒高发地区，应考虑采用聚合酶链反应检测确认给孕妇使用的血是否安全。

## Резюме

**Цель:**

Оценить сроки наступления основных событий при естественном течении инфицирования вирусом Зика.

**Методы:**

В феврале 2016 года мы провели поиск публикаций, в которых встречался термин «*Зика*», по базам PubMed, Scopus и Web of Science. На основе анализа данных публикаций мы приблизительно определили длительность инкубационного периода, время сероконверсии и длительность выделения вируса во внешнюю среду. Мы также приблизительно оценили риск получения донорской крови, содержащей вирус Зика.

**Результаты:**

Мы отобрали 20 статей о 25 пациентах с инфекцией, вызванной вирусом Зика. Медиана инкубационного периода при данной инфекции, по нашим оценкам, составила 5,9 дня (95% доверительный интервал, ДИ: 4,4–7,6), при этом у 95% лиц симптомы заболевания проявились в течение 11,2 дня (95% ДИ: 7,6–18,0) после заражения. Сероконверсия в среднем происходила спустя 9,1 дня (95% ДИ: 7,0–11,6) после заражения. В крови вирус обнаруживался в среднем на протяжении 9,9 дня (95% ДИ: 6,9–21,4). При отсутствии скринингового обследования риск получения донорской крови от инфицированного лица может быть приблизительно оценен как увеличение заражений вирусом Зика на 1 из 10 000 на каждый 1 из 100 000 человеко-дней. Скрининговое обследование на основании симптомов может снизить этот показатель на 7% (относительный риск, ОР: 0,93; 95% ДИ: 0,89–0,99), а скрининговое обследование по наличию антител — на 29% (ОР: 0,71; 95% ДИ: 0,28–0,88).

**Вывод:**

Ни скрининговое обследование по симптомам, ни скрининговое обследование по наличию антител инфекции вируса Зика не в состоянии существенно снизить риск того, что донорская кровь будет содержать этот вирус. Для определения безопасности донорской крови для переливания ее беременным женщинам в регионах с высокой заболеваемостью данной инфекцией следует обратиться к тестированию с помощью ПЦР.

## Introduction

In early 2016, the World Health Organization (WHO) declared a public health emergency of international concern because of the explosion in the number of people infected with the Zika virus in Central and South America and indications that the virus was responsible for an epidemic of microcephaly in Brazil.^1^ By 29 February 2016, at least half a million people in the Americas had been infected.^2^^,^^3^ Although the clinical disease is generally mild or asymptomatic,^4^ there is increasing evidence that Zika virus infection during pregnancy is linked to severe microcephaly in infants – there was a 10-fold increase in microcephaly cases in Brazil in the wake of the 2015 Zika virus epidemic.^5^ In adults, the infection has been linked to Guillain–Barré syndrome.^5^^,^^6^

The severity of these complications highlights the need to protect pregnant women from infection and to ensure that blood supplies remain safe, both in areas experiencing ongoing Zika virus transmission and in places with travellers returning from affected areas. There are concerns about potential transmission through blood transfusion because a large proportion of people infected with the virus remain asymptomatic,^4^ current diagnostic techniques are inadequate and the duration of viraemia and viral shedding are uncertain. In an outbreak in French Polynesia in 2013 and 2014, researchers found that 3% of asymptomatic blood donors were infected with the Zika virus and, in Brazil, several cases of possible viral transmission through blood transfusion were investigated in early 2016.^7^^,^^8^ As a result, some agencies, including WHO and the United States Food and Drug Administration, recommended deferring or halting blood donations from individuals within, or returning from, areas with active Zika virus transmission.^9^^,^^10^ Subsequently, Puerto Rico began importing blood components on 5 March 2016, though local donations resumed on 2 April 2016 after the Food and Drug Administration (FDA) approved an investigational nucleic acid test for the Zika virus.^11^

As temporary deferral or the banning of blood donations could result in severe shortages in blood supplies, research on the duration of viraemia and the time to antibody seroconversion is vital for quantifying the risk to blood supplies and for developing strategies for protecting them. Furthermore, better knowledge of the natural history of Zika virus infection, including the incubation period and infectious period, is essential for designing evidence-based surveillance systems and informing public health policy.^12^^,^^13^ Historically, estimates of the incubation period of even common diseases have been based on limited data. For instance, most of what we know about the incubation period of the respiratory syncytial virus is based on one observational and one experimental study, which involved fewer than 20 individuals in total.^14^^,^^15^ The situation does not necessarily improve as the prevalence of a disease increases because it becomes more difficult to establish the time range within which infection occurred. Previously, to make the best use of the limited data available, we developed an approach to estimating the incubation period and the distributions of other key variables in the natural history of an infectious disease from coarsely observed data.^14^^,^^16^^–^^20^ Using this approach, any case report that enables us to set bounds on the time of possible infection and the timing of an event of interest, such as symptom onset, seroconversion or viral clearance, can contribute to estimates of key variables in a statistically principled manner.

The aim of this study was to better characterize the natural history of Zika virus infection and to inform disease prevention, surveillance and blood supply safety strategies by applying an extension of this analytical approach to case reports collected through a systematic review of the literature. Using pooled data, we estimated the incubation period, the time to seroconversion and the duration of viral shedding from Zika virus infection.

## Methods

**Fig. 1:**
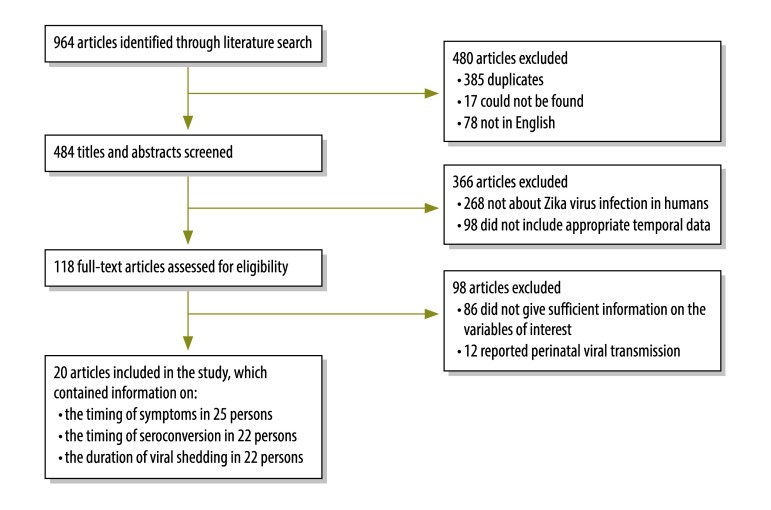
Flowchart showing the selection of studies on natural history of Zika virus infection, 1956–2016

Note: The full texts of 55 articles in French, Portuguese or Spanish were screened and none was found to contain relevant information.

We searched the PubMed, Scopus and Web of Science databases on 8 and 25 February 2016 (i.e. 7 and 24 days, respectively, after WHO’s declaration of a public health emergency of international concern) for publications containing the term *Zika* in any field. For our analysis, we included publications that provided information on: (i) the time of exposure to the Zika virus; (ii) the time of symptom onset; and (iii) the time of sample collection for, and the result (i.e. positive or negative) of, virological (e.g. by polymerase chain reaction or culture) or antibody Zika virus testing or both. Each publication was randomly assigned to two reviewers who independently screened titles and abstracts for relevance. We excluded publications that: (i) were not about the Zika virus; (ii) contained no data from humans; (iii) did not provide sufficient information to determine a bounded time of exposure; (iv) reported only perinatal viral transmission; or (v) were not in English. However, papers in French, Portuguese or Spanish that would have passed abstract screening had they been in English (i.e. 55 of 78 non-English articles) were also reviewed (Fig. 1). Then, two reviewers independently reviewed the full text of each publication to identify those with sufficient data for analysis. We contacted authors to obtain additional information when necessary. Discrepancies were resolved by discussion and consensus. The systematic review was conducted according to Meta-analysis of Observational Studies in Epidemiology group guidelines^21^ and Preferred Reporting Items for Systematic Review and Meta-Analyses guidelines,^22^ where applicable.

We abstracted the data necessary to estimate: (i) the incubation period of the Zika virus; (ii) the timing and duration of viral shedding; and (iii) the time to a positive serum antibody test result. In particular, we obtained information that enabled us to determine upper and lower bounds on the time of: (i) exposure to the Zika virus; (ii) symptom onset; and (iii) sample collection for virological or antibody testing. The exact timing of events was used when possible; otherwise bounds on the timing of an event were derived from the information available (e.g. the dates of travel to an endemic region). For virological and serological tests, the test result was recorded and specific immunoglobulin-M serological test results were noted, when available. We also recorded basic demographic details, the type of sample collected (e.g. blood or urine) and, when available, the mode of viral transmission.

The bounds for the time of Zika virus infection were the earliest and latest possible times of exposure consistent with the case report. When no latest exposure time could be determined – for example, the patient developed symptoms in an endemic area – it was assumed to be the latest possible time of symptom onset – the time of symptom onset was specified to the nearest day in most case reports. The earliest possible time of seroconversion was considered to be immediately after the last negative serological test and the latest possible time was immediately before the first positive test. If only a positive serological test was reported, the earliest possible time of seroconversion was considered to be the same as the earliest possible time of exposure; when only a negative test was reported, seroconversion was considered to have occurred after the time of testing. Similarly, the earliest possible time of viral clearance, which was defined as no detectable virus in blood, was the time of the last positive virological test and the latest was the time of the first negative test. Missing virological test results were treated in the same way as missing serological test results.

### Statistical analysis

For each observation of a time to a key event in the course of Zika virus infection (e.g. symptom onset, seroconversion or the end of viral shedding), we used the observed data to derive upper and lower bounds for the time of exposure and for the event. These censored observations were then used to fit separate distributions for each key event using an adaptation of previously described techniques.^14^^,^^16^ Briefly, Markov chain Monte Carlo methods were used to simultaneously fit the incubation period distribution (assuming a log-normal distribution) and the distributions of the time to immunoglobulin-M seroconversion (assuming a Weibull distribution) and the time to viral clearance (assuming a Weibull distribution) to the doubly interval-censored data. For the incubation period, we report the dispersion of the log-normal distribution as the exponential of the log-scale standard deviation – this metric was used by Sartwell to characterize the incubation period and has the property that 66% of incubation periods will lie between the values of the median ÷ dispersion and the median × dispersion.^23^ Given a time of infection, the times to symptom onset, seroconversion and viral clearance were considered to be independently distributed. The mean incubation period and the times within which 5, 25, 50, 75 and 95% of patients who will develop symptoms are expected to do so were estimated. Details and software published elsewhere.^24^

We estimated the impact of the distributions of key variables on the safety of blood supplies for a constant incidence rate. The number of blood donors with a possible Zika virus infection in every 100 000 in the absence of screening was calculated as the daily incidence of infection per 100 000 multiplied by the mean time to viral clearance. This estimate was adjusted for symptom-based screening using the mean time to symptom onset and by assuming that 80% of those infected remain asymptomatic.^25^ In addition, the effect of screening based on serological tests was estimated using the mean time to the first of either seroconversion or viral clearance, which were assumed to be independent: patients who experienced seroconversion would be successfully excluded from donation by screening and those who cleared the virus would no longer be infectious. Analyses were performed using the JAGS program version 3.3(GNU General Public License version 2) and the R statistical language (R Foundation, Vienna, Austria).^26^^,^^27^

## Results

The literature search identified 964 articles containing the term *Zika*, of which 118 were selected for full text review (Fig. 1). We contacted the authors for four articles with insufficient information, none of which was included in the analysis. Finally, we extracted data from 20 articles on 25 patients infected with Zika virus, most of whom were infected after 2008 (Table 1).^4^^,^^28^^–^^46^ Most affected individuals were American or European residents, none was a child and 14 were male. Data were available on the time of symptom onset for 25 individuals, on 49 virological tests on 22 individuals and on 62 serological tests on 22 individuals. Twenty-three were infected while travelling in an endemic area, one via sexual transmission and one through experimental infection.

**Table 1:** Characteristics of 25 reported patients with a Zika virus infection, worldwide, 1956–2016

First author of publication (year)	Patient’s characteristic								
	Age (years)	Sex	Place of origin	Probable place of Zika virus infection	Year of exposure to the virus	Length of exposure period (days)	Possible time to symptom onset, range (days)	Possible time to seroconversion, range (days)	Possible time to viral clearance from serum, range (days)^a^
Bearcroft (1956)^28^	34	Male	Europe	Nigeria	ND	< 1^b^	3–4	4–9	> 6^c^
Chen (2016)^29^	55	Male	United States	Costa Rica	2015	8	3–12	< 39	ND
Duffy (2009)^4^	ND	Female	United States	Yap Islands	2007	13	7–21	< 34	ND
Fonseca (2014)^30^	ND	Female	Canada	Thailand	2013	16	1–18	< 24^d^	26–28
Foy (2011)^31^	36	Male	United States	Senegal	2008	24	5–30	< 33	< 33^e^
	27	Male	United States	Senegal	2008	24	4–29	< 33	< 33^e^
	ND	Female	United States	United States^f^	2008	7	3–11	15–34	< 16^e^
Ginier (2016)^32^	51	Female	Switzerland	El Salvador, Guatemala	2015	14	3–18	< 24	> 23
Gyurech (2016)^33^	44	Female	Switzerland	Brazil	2015	1	4–17	19–23	< 23
Korhonen (2016)^34^	37	Male	Finland	Maldives	2015	183	1–185	ND	< 191^g^
Kutsuna (2014)^35^	30–35	Female	Japan	Bora Bora	2013–2014	10	5–16	< 21	< 21^h^
Kwong (2013)^36^	52	Female	Australia	Indonesia	ND	9	0–10	ND	13–24
Leung (2015)^37^	27	Male	Australia	Indonesia	ND	6^i^	2–9	ND	< 14^j^
Maria (2016)^38^	60–69	Female	France	Martinique	2015	22	1–24	< 28	ND
	20–29	Male	France	Brazil	2015–2016	8	0–9	< 17	ND
	50–59	Male	France	Colombia	2015–2016	29	0–30	31–37	ND^k^
Shinohara (2016)^39^	40–45	Male	Japan	Thailand	2014	7	1–9	10–14	> 10^d^
Simpson (1964)^40^	28	Male	Europe	Uganda	ND	76	0–77	< 78	> 2^c^
Summers (2015)^41^	48	Male	United States	Bolivia (Plurinational State of), Chile, Easter Island, Ecuador, French Polynesia, Hawaii, Peru	2013	34	0–35	< 45	ND
Tappe (2015)^42^	45	Female	Germany	Malaysia	2014	22	5–28	29–33	< 30
Tappe (2014)^43^	50–55	Male	Germany	Thailand	2013	12	0–12	< 22	< 22
Wæhre (2014)^44^	31	Female	Norway	Tahiti	2013	15	0–16	20–52	20–52^e^
Zammarchi (2015)^45^	60–65	Male	Italy	Brazil	2015	12	0–13	< 16	< 16
Zammarchi (2015)^46^	30–35	Female	Italy	French Polynesia	2013–2014	19	0–20	22–58	> 22
	30–35	Male	Italy	French Polynesia	2013–2014	19	0–20	22–56	< 23

ND: not determined.

^a^ Viral clearance was defined as no detectable virus in blood.

^b^ Inoculation of a volunteer with the Zika virus.

^c^ Viral shedding determined from mouse inoculation.

^d^ An equivocal test result was counted as a positive result.

^e^ Serum tested positive for the virus on polymerase chain reaction (PCR) testing but was negative on culture.

^f^ Probable sexual transmission.

^g^ Serum tested negative for the virus on PCR testing but urine tested positive on PCR testing at a later visit.

^h^ Serum tested negative for the virus on PCR testing but urine tested positive on PCR testing.

^i^ Possible transmission from a monkey bite or mosquito.

^j^ Serum taken from, and a swab of, the site of the monkey bite tested negative for the virus on PCR testing but a nasopharyngeal swab tested positive on PCR testing.

^k^ No sera tested; plasma and urine tested positive for the virus on PCR testing; at a later visit, plasma tested negative on PCR testing and urine and saliva tested positive on PCR testing.

**Fig. 2:**
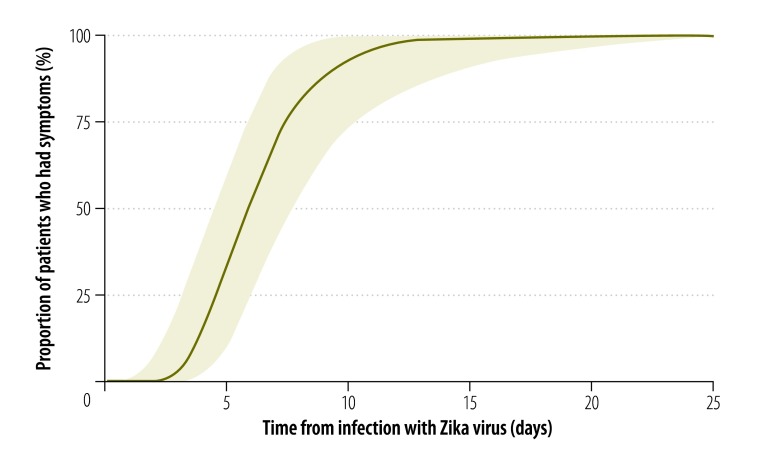
Zika virus incubation period, pooled analysis of cases, 1956–2016

Notes: An explanation of how time periods were derived is given in the methods section. The shaded areas indicate 95% credible intervals.

**Fig. 3:**
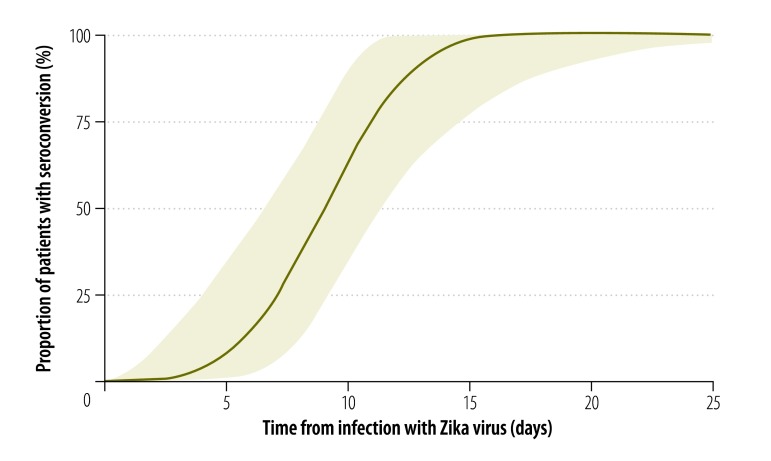
Zika virus seroconversion, pooled analysis of cases, 1956–2016

Notes: An explanation of how time periods were derived is given in the methods section. The shaded areas indicate 95% credible intervals.

**Fig. 4:**
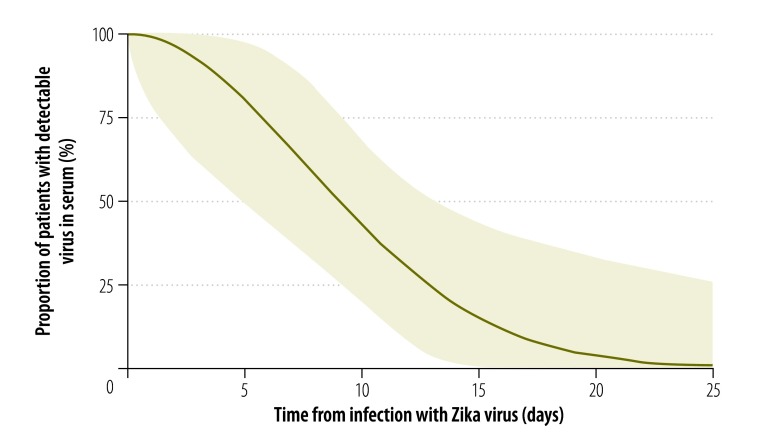
Zika virus clearance, pooled analysis of cases, 1956–2016

Notes: An explanation of how time periods were derived is given in the methods section. The shaded areas indicate 95% credible intervals.

We estimated the median incubation period of Zika virus disease to be 5.9 days (95% credible interval, CrI: 4.4–7.6), with a dispersion of 1.5 days (95% CrI: 1.2–1.9). Hence, 5% of symptomatic cases would be expected to develop symptoms within 3.2 days of infection (95% CrI: 1.7–4.6), 25% within 4.6 days (95% CrI: 3.1–6.0), 75% within 7.6 days (95% CrI: 5.8–10.4) and 95% within 11.2 days (95% CrI: 7.6–18.0; Fig. 2). The estimated mean time to seroconversion was 9.1 days (95% CrI: 7.0–11.6) after infection: 5% of cases would be expected to have detectable antibodies by 4.4 days (95% CrI: 1.3–7.0), 25% by 7.1 days (95% CrI: 4.0–9.2), 75% by 11.0 days (95% CrI: 8.7–14.6) and 95% by 13.7 days (95% CrI: 10.6–21.7; Fig. 3). The mean time to viral clearance was estimated to be 9.9 days (95% CrI: 6.9–21.4) after infection: 5% of cases would be expected to have no detectable virus by 2.4 days (95% CrI: 0.09–5.9), 25% by 5.8 days (95% CrI: 1.4–9.2), 75% by 12.7 days (95% CrI: 9.2–25.9) and 95% by 18.9 days (95% CrI: 13.6–79.4; Fig. 4).

Given that the estimated mean time to viral clearance from blood is 9.9 days, each 1 in 100 000 increase in the daily incidence of Zika virus infection would be associated with an increase in the proportion of infected blood donors of 9.9 per 100 000 (95% CrI: 6.9–21.4) if no screening were performed. Refusing donations from people with recent symptoms of a possible infection would decrease this risk by only 7% (relative risk, RR: 0.93; 95% CrI: 0.89–0.99), assuming that 80% of individuals with Zika virus infections are asymptomatic and given that those who develop symptoms are infectious but asymptomatic for an average of 5.9 days – here it is assumed that the Zika virus can be transmitted via blood from the moment of infection. Serological screening would reduce the risk by 29% (RR: 0.71; 95% CrI: 0.28–0.88) but improve the safety of blood supplies only marginally. In settings where the risk is solely from imported Zika cases, ensuring blood supply safety is easier. We estimated that, by 23.4 days (95% CrI: 14.3–154.3) after infection, 99% of cases would no longer have detectable virus in their blood. For these estimates, we assumed that a blood donation would be safe if there was no detectable virus in the donor’s blood. However, in four reported cases, a saliva, nasal or urine sample tested positive for the virus even though it was no longer detectable in blood (Table 1). Blood donation may, therefore, still pose a risk. Although few data were available on viral clearance in these fluids, we estimated that the latest positive saliva, nasal or urine test took place a mean of 12.0 days (95% CrI: 10.1–18.2) after infection.

## Discussion

In June 2016, WHO reported that the incubation period of the Zika virus was uncertain but likely to be “a few days”.^47^ Similarly, the United States Centers for Disease Control and Prevention stated that the period was unknown but probably “a few days to a week” and the European Centre for Disease Prevention and Control estimated it was 3 to 12 days.^48^^,^^49^ Our analysis indicates that the incubation period is around 6 days and gives an estimate of the remaining uncertainty. In addition, we provide estimates of the times to seroconversion and viral clearance. Knowledge of these key variables in the natural history of Zika virus infection is important for designing and evaluating screening and surveillance protocols, as we illustrated in our analysis of screening for Zika virus infection in blood donors. Although the risk to blood supplies is quite low, it is proportional to the incidence of infection, which is hard to measure because many cases are asymptomatic. Screening for symptoms is important but only a direct antigen test can reduce risk. Our analysis indicates that antibody tests could reduce the risk of contaminated blood supplies by around 30%.

In practice, many countries and organizations recommend deferring blood donation until 28 days after the resolution of symptoms or after the time of a positive serological or virological test result for an asymptomatic individual, as recommended by WHO^10^ – the United States FDA recommended 28 days, the Brazil Ministry of Health recommended 30 days and the Canadian Blood Services recommended 21 days.^9^^,^^50^^,^^51^ Our analysis indicated that well over 99% of people with a Zika virus infection will have no detectable virus in their blood after this period. However, since most laboratory testing will take place because symptoms are present, these recommendations essentially concern symptomatic screening, which we estimate will reduce the probability that a blood donor will have a Zika virus infection by less than 10%. Since it may not be practical to stop blood donations until a Zika virus epidemic has passed, countries may consider virological (i.e. nucleic acid) testing, particularly of blood for use in pregnant women. However, nucleic acid testing may not be perfect: we found one case in which a negative test result was followed by a positive test result, though this was in the context of perinatal transmission.^52^ In settings where the risk to blood donations comes solely from imported Zika cases, ensuring safety is far easier. We estimated that 99% of patients would no longer have detectable virus in their blood 23.4 days after infection. Although this figure was based on only a few observations, it can guide the deferral of blood donations: for example, donations could be accepted only 300 days after travel to a region where the Zika virus is endemic – this period is more than twice the upper 95% credible interval for viral clearance from 99% of affected patients.

Our study highlights the need for a highly specific, simple and rapid Zika virus antigen test. As well as for screening donated blood, an antigen test could be used to monitor the incidence of Zika virus infection, to help provide advice for women considering becoming pregnant and to identify pregnant women at risk of a poor clinical outcome. In addition, saliva or urine could be used in diagnostic tests and research into the relationship between the presence of the virus in bodily fluids and the risk of disease transmission is also needed. Knowledge of the time between infection and the virus becoming detectable in various fluids is essential for ascertaining the ability of antigen testing to ensure the safety of blood and organ donations. Moreover, knowledge of the incubation period can help clinicians determine whether Zika virus infection should be considered in the differential diagnosis of febrile patients who have recently travelled abroad and a good estimate of the time to seroconversion can help optimize the timing of confirmatory testing. Knowing the time to viral clearance after a potential infection or exposure can indicate when it may again be safe to become pregnant. However, the time to clearance from seminal fluid remains unknown. Our analysis provides only a first step in determining the values of key variables in Zika virus infection as the small number of cases included means that there are substantial uncertainties and a potential for bias. Investigators should continue to collect data to refine and update our estimates and to provide information on other key variables in the disease’s natural history, such as the latent period (i.e. the time between being infected and becoming infectious). To assist that process, all our data and the analysis software we used are freely available to enable other investigators to contribute to this work or apply our methods to their own data.^24^

Our analysis necessarily involved several assumptions because the published data were not collected to assess key variables in Zika virus infection. First, we assumed that virological testing of blood or sera had a sensitivity of 100% for detecting a Zika virus infection – however there is evidence that viral shedding can continue longer in urine and other bodily fluids and the virus may persist in the blood below the limit of detection. We also assumed that the time to seropositivity for the Zika virus was independent of previous infections – however it is probable that people who have had a prior flavivirus infection may seroconvert more quickly. Consequently, it is possible we overestimated the time to seroconversion because the majority of cases in our analysis were travellers returning to countries where flaviviruses were not endemic. Another limitation is that the majority of our data were from people presumed to have been infected through mosquito bites. The timing of key events may differ for other routes of infection (e.g. perinatal or sexual). Furthermore, all our cases were symptomatic – the time to seroconversion or viral clearance may be different in asymptomatic individuals and it is possible that cases reported in the literature may have been more severe than usual. Nevertheless, the principle limitation of our analysis was the small number of cases.

Despite these limitations, our analysis provides the most detailed, quantitative estimates to date of the timing of key events in the natural history of Zika virus infection. Our findings can help guide disease surveillance in both endemic areas and in returning travellers and can underpin research into the basic features of this pathogen.
